# One-Year Changes in Bioelectrical Impedance Data in Adolescent Athletes

**DOI:** 10.3390/nu16050701

**Published:** 2024-02-29

**Authors:** Marcus Vinícius de Oliveira Cattem, Camila E. Orsso, Maria Cristina Gonzalez, Josely Correa Koury

**Affiliations:** 1 Nutrition Institute, Rio de Janeiro State University, Rio de Janeiro 20550-013, Brazil; mv_cattem@hotmail.com; 2 Human Nutrition Research Unit, Department of Agricultural, Food and Nutritional Science, University of Alberta, Edmonton, AB T6G 2P5, Canada; orsso@ualberta.ca; 3 Post-Graduate Program in Nutrition and Foods, Federal University of Pelotas, Pelotas 96010-610, Brazil; cristinagbs@hotmail.com

**Keywords:** bioelectrical impedance analysis, adolescents, athletes

## Abstract

Raw bioelectrical impedance (BI) data and vector analysis (BIVA) have been used to evaluate fat-free mass (FFM) cross-sectionally in adolescent athletes; however, there have been no longitudinal studies about it. This study aimed to assess the magnitude of changes in raw BI data (resistance [R], reactance [Xc], and phase angle [PhA]), BIVA, and FFM in adolescent athletes (*n* = 137, 40% female). BI data were collected using a single-frequency device at baseline and after one year of sports practice. Baseline chronological age categorized the participants (11, 12, or 13 years [y]). In females, Xc/H increased (13 to 14 y, *p* = 0.04) while R/H decreased in all age groups (*p* = 0.001). PhA (11 to 12 y, *p* = 0.048) and FFM (11 to 12 y and 12 to 13 y groups *p* = 0.001) increased and showed the lowest magnitude of changes in the 13 to 14 y group (*p* = 0.05). In males, Xc/H decreased (11 to 12 and 12 to 13 y groups, *p* = 0.001) with a higher magnitude of changes in the 13 to 14 y group (*p* = 0.004); R/H decreased (*p* = 0.001); FFM increased in all groups (*p* = 0.001); however, no magnitude of changes was observed. PhA increased in the 13 to 14 y group (*p* = 0.004). BIVA showed no differences among ellipse distances in females. In males, a high distance was observed in the 11 to 12 y group. “Time interval” influenced PhA and Xc/H in the female group and R/H and Xc/H in the male group. “Initial age” and “time interval” influenced the increase in PhA in the male group. Raw BI data and BIVA patterns can detect the magnitude of the changes in a sex-dependent manner.

## 1. Introduction

Sports training in childhood and adolescence has a positive effect on various body functions, including skeletal muscles, cardiovascular health, blood circulation, neurological functions, and metabolic systems. Cardiovascular and muscular fitness are related to improvements in general health. Furthermore, sports training can positively affect body composition by increasing muscle mass and bone density while decreasing fat mass in adolescent athletes [1,2]. Additional factors, such as sex, age, hormonal, nutritional, and maturity status, can also impact body composition [3,4,5]. Body composition, particularly skeletal muscle mass (SMM), often relates to muscle strength and physical capabilities. In two-compartment methods, such as bioelectrical impedance analysis (BIA), fat-free mass (FFM = residual mass, skeletal muscle mass, and bone mineral content) has been used as an SMM marker [6]. Predictive equations for skeletal muscle mass (SMM) have been developed using BIA data from adult individuals [7]. However, there are currently no SMM equations specifically designed for adolescents. While FFM–BIA predictive equations for adolescents are available [8,9,10,11,12], only one predictive equation that considers biological maturity has been identified for adolescent athletes [8].

BIA is a non-invasive, low-cost, and portable method to estimate body composition, including total body water (TBW), intra- and extracellular water (ICW and ECW) from electrical body proprieties of resistance (R), and reactance (Xc) [13,14,15]. BIA relies on predictive equations for body composition that depend on individual characteristics such as sex, age, and maturity status [8,14,15], as well as on device specifications. The accuracy may be compromised when applied to a population with different characteristics or when devices with different technologies are used [16,17,18]. However, raw BIA variables can be a viable alternative because these measures are not subject to population-specific constraints such as the phase angle (PhA). PhA is calculated using the Xc and R ratio and interpreted as an index of membrane stability, integrity, and nutritional status [15,19]. PhA has been used in clinical practice associated with positive clinical outcomes and in sports as a possible proxy of health, physical performance, and muscle strength [13,20,21,22,23,24,25,26,27]. 

Bioelectric impedance vector analysis (BIVA) is based on a qualitative and semiquantitative analysis of raw bioelectrical data plotted in ellipse graphs [28,29,30,31,32,33,34,35]. It can be used in physically active adults and adolescents to assess BCM and TBW without using predictive equations [13,25,35]. Cross-sectional studies in children and adolescents have shown that chronological age advancing, and biological maturity can lead to increased PhA values, consistent with the increase in BCM related to growth. In addition, the shorter vector length (Z/H) is associated with the increase in TBW values [8,21,25,36,37,38,39,40,41,42]. Nevertheless, distinguishing whether the BCM and TBW adaptation stems from biological maturity or results from physical training remains challenging [1,43,44]. Therefore, monitoring changes in BIVA must be carried out periodically.

Cross-sectional studies have demonstrated that physiological and morphologic changes that occur during adolescence lead to bioelectrical properties different from those of adult athletes [13]. A study on adolescent athletes showed that PhA and BIVA ellipses are, possibly, influenced by routine intense training, which causes changes in the functional and hydration parameters. The magnitude of these changes in adolescents may depend on the sports modality practiced [13]. In addition to that, raw bioelectrical data in healthy adolescents can be affected according to biological maturity status [21,25,36]. In Italian female adolescents between 10 and 15 years of age, sexual maturity seemed more important than differences in chronological age since higher raw bioelectrical data were found regarding maturity than age differences [21]. In male adolescent football players, it was observed that bone age and zinc erythrocyte contribute to PhA and that BIVA was influenced by skeletal maturity status [36].

Unlike cross-sectional studies, which focus on differences between groups at a single time point, longitudinal studies typically aim to determine the magnitude of changes over a period. Longitudinal studies can also define vector migration trends more clearly. Orsso et al. [45] have emphasized the importance of longitudinally estimating body composition using BIA among children and adolescents, especially for longer follow-up periods. FFM predictive equations, developed through cross-sectional studies using reference methods, have not been tested longitudinally in healthy adolescents or adolescent athletes. BI data and BIVA have been used in synchronized swimmer adults (20.9 ± 1.9 years old), and BIVA has detected changes in body fluid and cellularity after a 13-week physical training program [46]. To our knowledge, this is the first study that aims to identify the magnitude of changes in raw BIA data, in BIVA, and in FFM in adolescents after one year of sports participation.

## 2. Materials and Methods

### 2.1. Study Design

This was a longitudinal study, with data collected at baseline (T0) from August to December 2018 and after one year during the same months (T1). 

### 2.2. Participants

Adolescents and parents/guardians agreed to participate after we fully explained the study objectives and assessment methods. This study was approved by the Ethics Committee of the Pedro Ernesto Hospital (CEP/HUPE 1.020.909-2018). 

This study was intentionally limited to healthy adolescent athletes to minimize inter-participant variance and maximize the applicability of using BIA and BIVA. Throughout the study, we did not experience any loss to follow-up. 

All the data were collected at a sports-oriented public school in the central region of Rio de Janeiro, Brazil. This elementary full-time school offers 100 min of daily sports training (swimming, judo, badminton, athletics, soccer, volleyball, and table tennis).

The students can choose a different sport to practice each week. This way, they can decide which sport fits them best in the future. The adolescents were classified as athletes because they participated in physical training and skill development and were engaged in competition events [5]. 

Female sexual maturation was self-reported as the age at menarche. The participants who had already begun their menstrual cycle were classified as sexually mature and those who had not were classified as sexually immature. In male adolescents, given the lack of reliable markers for maturity, biological maturity was estimated to be >13 years [1,25,41,42,43]. 

### 2.3. Anthropometric Measurements

Weight was measured with a portable scale to the nearest 0.1 kg (Filizola, Brazil) and height with a stadiometer to the nearest 0.5 cm (Sanny, Brazil). Body mass index was calculated (BMI (kg/m^2^) = weight (kg)/(height (m))^2^). The nutritional status of the participants was evaluated using BMI-for-age and height-for-age z-scores according to WHO [47].

### 2.4. Bioelectrical Impedance Analysis

Bioelectrical parameters (R and Xc, in ohms) were measured with a single-frequency tetrapolar impedance analyzer (RJL, model 101 Quantum, RJL Systems, Clinton Township, MI, USA) using a current of 800 μA at an operating frequency of 50 kHz. Whole-body impedance measurements were taken using the supine positioning of outer and inner electrodes on the right wrist and ankle, previously cleansed with alcohol. To avoid disturbances in fluid distribution, participants were placed in a thermoneutral environment (25 °C) and were instructed to abstain from foods and liquids for at least 4 h and from caffeine and intense physical activity for at least 24 h before the BIA measurements. 

All metals and conductive accessories were removed before each BIA measurement. Female participants underwent data collection at a time point outside of their menstrual periods. The BIA analyzer was checked with a predetermined impedance circuit calibration (R = 500 ohms; 0.9% error) and the components inside the bioimpedance analyzer, such as signal generator, sensing apparatus, and electrical interference, were tested before each test session. 

BIA data was used to predict FFM (kg) using the equation [3.474 + 0.459 H(m)^2^/R(Ω) + 0.064 weight (kg)]/[0.769 − 0.009 age (years) − 0.016 sex (0 if female, 1 if male)] [9]. Horlick et al.’s [9] equation was chosen to predict FFM because it was shown with acceptable agreement with DXA when tested in Brazilian adolescent athletes [8]. Furthermore, although one study had developed an FFM predictive equation using a single-frequency BIA in adolescent athletes [8], it was impossible to apply this equation in the present study because we did not collect skeletal data, a variable considered in this predictive equation. FFM index (FFMI) was calculated using the equation FFMI (kg/m^2^) = FFM (kg)/H(m)^2^ [48].

PhA was calculated using the equation of arctangent (Xc/R) × 180°/π [19]. Additionally, BIVA analysis was performed using R and Xc adjusted by height [34]. Sample size absolute differences (d) dR/H and dXc/H determined the position and size of the ellipses. Correlation between dR/H and dXc/H determined the ellipsoidal form. Changes in R/H were related mainly to fluids (inverse relation), and in Xc, to cellularity (direct relation). 

### 2.5. Statistical Analyses

The sample size had been determined a priori using statistical software (G*Power 3.1.9.7 Stuttgart, Germany) assuming an effect size of 0.5, α of 0.05, and β of 20%. Forty-five participants had been estimated by sex. The normality of the data distribution was tested using the Kolmogorov–Smirnov test, considering each group. As data were normally distributed, continuous variables were expressed as means ± standard deviations (SDs). The paired *t*-test was used to compare T0 and T1 changes within each age group and sex.

All analyses were performed separately for each sex and age group. Age groups are presented as the participants’ ages at T0 and the participants’ ages at the one-year follow-up (T1). Our participants were classified into three different groups based on their ages at T0 and T1 as follows: group 1 = 11 to 12, group 2 = 12 to 13, and group 3 = 13 to 14 years.

Two-way ANOVA was used to test the effects of initial chronological age (initial “age”, factor 1) and age after a one-year time interval (“time interval”, factor 2) on bioelectrical data. Factor 1 represented accumulated growth and development up to the start of the study (T0), and factor 2 was interpreted as growth, development, and maturation of the participants accumulated during the study (T1). Furthermore, confidence ellipses for paired data and the respective Hotelling T^2^, Mahalanobis distances (D), F, and *p* values were calculated to analyze longitudinal changes in impedance vectors from T0 to T1 stratified by sex. 

All *p*-values < 0.05 were considered statistically significant. All statistical analyses were performed using STATISTICA 10 software (Stat Soft. Inc., Tulsa, OK, USA) or BIVA software 2002 [49].

## 3. Results

A total of 82 male and 55 female adolescent athletes participated in the study, and no participants were lost to the follow-up at T1. All participants presented normal height-for-age and BMI-for-age (15th and 85th percentiles) [47]. Table 1 presents the sports participants who self-reported practicing at T0. 

In females, it was observed that Xc/H increased in the 13-to-14-year group whereas R/H decreased in all age groups. PhA increased in the 11-to-12-year group, and FFM increased in all age groups. PhA (7.3%) and FFM (2.7%) presented the lowest magnitude of changes in the 13-to-14-year group. There was an increase in BMI values in the 11-to-12 and 12-to-13-year groups; however, BMI classification remained unchanged regardless of age, classified as normal weight at both T0 and T1 (Percentiles 74.8 and 75.2, respectively). Although FFM increased in these groups (11-to-12 and 12-to-13-year groups), FFMI did not change over the year, indicating that the increase in FFM did not surpass the gain in height. In males, it was observed that Xc/H decreased in the 11-to-12 and 12-to-13-year age groups with a higher magnitude of changes in the 13-to-14 year-group (1.9%); R/H decreased whereas FFM increased in all groups, and no magnitude of changes was observed; PhA increased in the 13-to-14-year group with a magnitude of changes of 12.3%. BMI value increased only in the 12-to-13-year group. However, BMI classification remained unchanged, regardless of age, classified as normal weight at both T0 and T1 (Percentiles 82.1 and 78.6, respectively). FFMI increased in all males, indicating a more pronounced increase in FFM than in height (Table 2).

**Figure 1:**
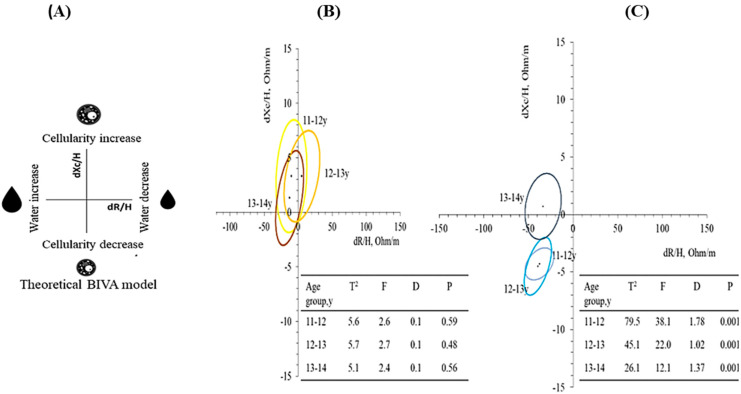
Theoretical BIVA model resistance [R] and reactance [Xc] changes (delta [d]), normalized by the height (H, meter) (**A**). Paired confidence ellipses of classic BIVA in female (**B**) and male (**C**) adolescents according to age groups after one year. The differences between the mean impedance vectors in the different age groups of female and male adolescents were determined using the Hotteling T^2^ test. The distances [D] between ellipses were calculated using the Mahalanobis test, which is a descriptive statistic that provides a relative measurement of data point distances (residual) between vectors. The *p*-value [P] considers the distance to the graph’s origin (dXc/H = dR/H = 0). Significant values indicate changes in bioelectrical data and correlated body composition.

One-year changes in bioelectrical vectors were significant only in males, regardless of age group. Figure 1A demonstrates theoretical BIVA model resistance [R] and reactance [Xc] changes (delta [d]), normalized by the height (H, meter) [49]. In female adolescents, 95% paired confidence ellipses for all age groups covered the null vector (Figure 1B). In contrast, male adolescents’ confidence ellipses differed among all age groups (*p* < 0.001) with a different pattern of migration in the 13 to 14 y group, related to the increase in PhA (Figure 1C). 

Table 3 shows the effect of “initial age” (T0) and “time interval” (after one year) on BIA raw data in female and male adolescents. In the female group, only the “time interval” influenced the changes in the PhA (*p* = 0.002) and the Xc/H ratio (*p* < 0.001). However, in the male group, the “initial age” influenced the changes in the R/H ratio (*p* = 0.002), Xc/H (*p* = 0.011), and Z/H (*p* = 0.002); the “time interval” influenced the changes in the R/H ratio (*p* < 0.001), Xc/H (*p* = 0.025), and Z/H (*p* < 0.001); and the interaction of “initial age” and “time interval” only influenced the PhA change (*p* = 0.035).

## 4. Discussion

This study identified the magnitude of changes in raw BI data, BIVA, and FFM in adolescents after one year of participating in sports, considering sex and age. In the female group, BIVA presented overlapped ellipses, indicating no significant differences in raw BI data according to age. Furthermore, PhA and Xc/H changes were influenced by “time interval” (considered as growth), indicating high cellularity and BCM increase. On the other hand, the male group showed a shortening in R/H (all age groups) and in Xc/H (11 to 12 y and 12 to 13 y groups), which indicates an increased TBW, and a decrease in BCM. 

Most studies have used BMI to assess nutritional status. However, these studies cannot clarify whether the associations were from the influence of growth on fat mass, FFM, or both [1,43]. In the present study, after one year, BMI changes classified all participants as having normal weights according to WHO reference curves [47]. In order to overcome the limitations of BMI, FFMI was calculated, indicating the maintenance of FFM for female athletes and an increase in FFM for male athletes, following the expected FFM increases predicted for adolescents [1].

Sex differences in FFM and the corresponding changes during puberty have been observed [1,43]. FFM remains comparable between the sexes during middle childhood (6–12 years of age). However, in puberty, males accrue approximately one kilogram more in absolute FFM than females [50], and for a longer period, males acquire more FFM than females [1,43,45]. This trend is similar to that observed in the present study, in which female adolescents (11 to 12 y and 12 to 13 y age groups) and male adolescents (11 to 12 y, 12 to 13 y, and 13 to 14 y age groups) showed an increase in FFM. Horlick et al.’s [9] BIA FFM predictive equation was used in the present study because the previous study showed it was adequate for adolescent athletes [8]. However, this result must be viewed with caution since BIA FFM predictive equations can lead to misinterpretation [51]. For this reason, BIVA must be carried out.

BIVA utilizes raw BI data to overcome the limitations of predictive equations. There are two approaches to BIVA: classic BIVA [33,34] and specific BIVA [29,30,31,32,52]. Classic BIVA has been suggested to be unable to differentiate individuals with different proportions of fat mass, as observed in elderly individuals [53] and male elite youth soccer players [40]. In a study with North American children and adolescents, body fat percentage was found to be positively associated with age among female adolescents and with FFMI among male adolescents [30]. In the present study, classic BIVA was used, and for this reason, fat mass was not calculated.

In a cross-sectional study with male youth soccer players, classic BIVA identified a shortening of R/H and Xc/H values in “earlier maturity” adolescents compared to “late maturity” [40]. Similar results were observed in the present study, in which R/H and Xc/H values were lower at T0 (baseline) in the mature group (13 to 14 years) compared to the immature group (11 to 12 years). After one year of sports practice, R/H values decreased in all male age groups whereas Xc/H values decreased only in the 11 to 12 and 12 to 13 y age groups, increasing in the 13 to 14 y age group. A decrease in R/H indicates a body fluid improvement that may be linked to high TBW and FFM values while the increase in Xc/H is related to growth and, consequently, high cellularity [40,46], consistent with the number of mature male adolescents in this age group. These findings are commonly observed in athletes who have relatively high values of lean body mass, a surrogate marker of muscle mass, muscle glycogen reserves, and plasma volume [45,54]. In the present study, PhA increased only in the mature group (13 to 14 y) after one year of sports practice, which is consistent with the simultaneous increase in TBW (fluid improvement) and BCM (higher cellularity). These results are related to development and growth, leading to an increase in FFM in all age groups and reinforcing that longitudinal BIVA is influenced by maturity status, according to other cross-sectional studies [25,41,42].

The relationship between PhA and exercise benefits has been studied in adults. Ballarin et al. [55] highlighted that PhA is correlated with physical fitness tests related to muscle strength. Additionally, it is a significant predictor of upper- and lower-limb musculoskeletal strength in young adults (24.2 ± 3.0 years). However, in adolescents, systematic reviews have shown limited and partial evidence that changes in PhA over the first two decades of life reflect modifications in the FFM. This suggests that more studies are needed to confirm PhA as a relevant marker of nutritional status in adolescents [26,29]. It should be noted that all the studies were cross-sectional, meaning they were based on observations from data collected just once in different groups, differently from longitudinal studies, which aim to determine the magnitude of changes over a period. In the present longitudinal study, “time interval” influenced, in females, PhA and Xc/H, and in males, R/H, Xc/H, Z/H, and PhA, and this was also influenced by “initial age”. These results corroborate the relationship between PhA and raw BI data with growth in healthy adolescent athletes.

Studies on BIVA in female adolescent athletes are scarce. Only one cross-sectional study has observed a negative association between Xc/H values and chronological age in Brazilian female adolescent athletes [25]. Studies have also observed progressive shortening vectors in Italian children and adolescents (2–15 y) of both sexes [41], in Italian female adolescents (10–15 y) [21], and in Brazilian female adolescents (11–12 y) [42]. These results may relate more to maturity status than chronological age [21].

Higher PhA values and shorter vectors have been found in post-menarche female adolescents when compared to pre-menarche ones [21]. In the present study, comparing the female groups based on sexual maturity proved challenging due to the limited representation of sexually immature females across all age groups. Possibly for this reason, no difference in vector length was observed after one year of sports practice. Nonetheless, this one-year longitudinal assessment revealed higher Xc/H values in the 13 to 14 y age group, where all participants were in the post-menarche stage. The PhA changes were higher in the 11 to 12 y group (58% sexual maturity), possibly due to greater variation in weight, height, and FFM compared to the 13 to 14 y group. BIVA showed overlapped ellipses, after one year of sports practice and growth, indicating no significant differences in bioelectrical vectors considering chronological age.

In the present study, there were some limitations, such as the absence of a reference method to identify biological maturity in male adolescents, the small number of sexually immature female adolescents, and the lack of a highly accurate method, such as DXA, to test the agreement of FFM changes occurring over time. Another important point to be considered is the small sample size after stratification by sex and chronological age, which are crucial in characterizing adolescents. 

The strengths of the present study were the following: no segment loss for all adolescent athletes for one year and practice regular sports under similar conditions. Moreover, this study highlighted the practical utility of employing raw BI data for changes in FFM measurement after one year and BIVA for the analytical interpretation of R and Xc adjusted by height. Taken together, they may enhance the comprehension of physiological changes during adolescence. For dietitians and physical trainers overseeing adolescent athletes, integrating BIVA into their monitoring protocols facilitates the assessment and monitoring of changes in body composition throughout their training programs. This integration not only aids in understanding the impact of physical training on physiological parameters but also supports tailored interventions to optimize athletes’ performance and overall health during this critical developmental phase.

## 5. Conclusions

Our results emphasize the applicability of raw BI data and BIVA ellipse patterns that can detect the magnitude of changes in a sex-dependent manner. Their integrated evaluation might contribute to identifying risks and planning training schedules and nutrition interventions for adolescent athletes.

## Figures and Tables

**Table 1:** Self-reported sport modalities at baseline (T0).

	Female *n* [%]	Male *n* [%]
Athletics	12 [23]	20 [24]
Soccer	8 [16]	21 [26]
Volleyball	11 [21]	20 [24]
Swimming	6 [12]	7 [8.5]
Table tennis	8 [16]	8 [10]
Handball	6 [12]	6 [7.5]

**Table 2:** Anthropometric, bioelectrical impedance raw, and body composition data, stratified by sex and age groups at baseline (T0) and after one year (T1) of practiced sports.

		Female			Male		
		11 to 12y	12 to 13y	13 to 14y	11 to 12y	12 to 13y	13 to 14y
		*n* = 16	*n* = 23	*n* = 16	*n* = 25	*n* = 43	*n* = 14
	T0	11.18 ± 0.26	12.32 ± 0.34	13.22 ± 0.32	11.27 ± 0.24	12.36 ± 0.29	13.41 ± 0.31
Age (years)	T1	12.25 ± 0.21	13.01 ± 0.22	14.19 ± 0.29	12.35 ± 0.27	13.03 ± 0.35	14.20 ± 0.34
	*p*-value	0.001	0.001	0.001	0.001	0.001	0.001
Weight (kg)	T0	48.3 ± 12.7	48.0 ± 10.9	52.9 ± 6.5	45.0 ± 12.5	47.9 ± 10.1	53.3 ± 9.6
	T1	53.5 ± 11.9	53.7 ± 12.4	55.4 ± 6.1	51.7 ± 13.4	54.8 ± 10.8	58.7 ± 10.1
	*p*-value	0.001	0.001	0.001	0.001	0.001	0.001
	Change	5.2 ± 2.7 ^a^	5.7 ± 3.2 ^a^	2.4 ± 2.3 ^b^	6.7 ± 4.1	6.9 ± 2.8	5.4 ± 3.4
	%	11.9	12.1	4.8	15.6	14.8	10.5
Height (cm)	T0	152.4 ± 7.0 ^a^	156.5 ± 6.7 ^a,b^	160.4 ± 6.7 ^b^	149.6 ± 8.3 ^a^	154.7 ± 7.4 ^b^	161.3 ± 7.9 ^c^
	T1	157.3 ± 6.5	159.8 ± 6.2	162.3 ± 6.7	157.8 ± 9.6 ^a^	163.2 ± 7.2 ^b^	168.4 ± 7.6 ^b^
	*p*-value	0.001	0.001	0.001	0.001	0.001	0.001
	Change	4.9 ± 2.1 ^a^	3.3 ± 2.7 ^a,b^	1.9 ± 1.2 ^b^	8.2 ± 2.7	8.5 ± 3.0	7.1 ± 2.7
	%	3.3	2.2	1.2	5.5	5.5	4.5
	T0	20.6 ± 4.2	19.4 ± 3.4	20.6 ± 2.7	19.8 ± 3.7	19.9 ± 3.2	20.2 ± 2.7
BMI (kg/m^2^)	T1	21.5 ± 3.8	20.9 ± 3.8	21.1 ± 2.4	20.5 ± 3.6	20.4 ± 3.2	20.6 ± 2.9
	*p*-value	0.005	0.001	0.102	0.061	0.002	0.288
	Change	0.9 ± 1.1 ^a,b^	1.4 ± 0.9 ^a^	0.4 ± 1.0 ^b^	0.7 ± 1.8	0.6 ± 1.1	0.4 ± 1.3
	%	4.9	7.4	2.5	4.0	3.1	2.0
R/H (Ω/m)	T0	414.1 ± 67.1	405.7 ± 47.1	386.5 ± 26.5	414.6 ± 63.7 ^a^	383.8 ± 58.2 ^b^	368.4 ± 49.5 ^b^
	T1	404.3 ± 74.3	393.2 ± 46.2	392.1 ± 54.0	376.8 ± 67.1 ^a^	344.3 ± 52.2 ^b^	334.8 ± 46.3 ^c^
	*p*-value	0.239	0.133	0.556	0.001	0.001	0.001
	Change	−9.8 ± 32.1	−12.5 ± 38.4	5.6 ± 37.1	−37.8 ± 30.6	−39.5 ± 39.0	−33.6 ± 26.1
	%	−2.4	−2.7	1.2	−9.2	−9.8	−8.9
Xc/H (Ω/m)	T0	41.1 ± 4.2	42.3 ± 5.7	41.9 ± 3.7	45.3 ± 7.0 ^a^	42.1 ± 7.0 ^a,b^	38.9 ± 5.2 ^b^
	T1	44.4 ± 7.5	43.7 ± 6.8	45.2 ± 6.2	41.0 ± 7.3	37.6 ± 5.3	39.6 ± 5.9
	*p*-value	0.092	0.448	0.040	0.001	0.001	0.517
	Change	3.3 ± 7.3	1.3 ± 8.1	3.3 ± 5.9	−4.3 ± 2.6 ^a^	−4.5 ± 6.4 ^a^	0.7 ± 3.7 ^b^
	%	8.4	4.5	8.2	−9.7	−9.5	1.9
Z/H (Ω/m)	T0	416.2 ± 67.1	407.9 ± 47.2	388.7 ± 26.6	417.1 ± 64.0	386.2 ± 58.4	370.5 ± 49.5
	T1	406.8 ± 74.1	395.7 ± 46.4	394.7 ± 54.0	379.1 ± 67.3	346.4 ± 52.3	337.2 ± 46.2
	*p*-value	0.260	0.142	0.530	0.001	0.001	0.001
	Change	−9.4 ± 32.0	−12.2 ± 38.6	6.0 ± 37.1	−38.0 ± 30.6	−39.8 ± 39.1	−33.3 ± 25.9
	%	−2.3	−2.6	1.3	−9.2	−9.8	−8.8
PhA (°)	T0	5.74 ± 0.57 ^a^	5.98 ± 0.64 ^a,b^	6.20 ± 0.46 ^b^	6.3 ± 0.6	6.3 ± 0.7	6.1 ± 0.7
	T1	6.40 ± 1.37	6.35 ± 0.85	6.64 ± 0.93	6.2 ± 0.6 ^a^	6.3 ± 0.7 ^a^	6.8 ± 1.1 ^b^
	*p*-value	0.048	0.120	0.084	0.833	0.926	0.007
	Change	0.7 ± 1.2 ^a^	0.4 ± 1.1 ^b^	0.4 ± 0.9 ^b^	0.0 ± 0.5 ^a^	0.0 ± 0.8 ^a^	0.7 ± 0.9 ^b^
	%	11.5	7.4	7.3	−0.1	0.4	12.4
FFM (kg)	T0	36.2 ± 6.3	37.3 ± 5.1	40.1 ± 2.6	36.1 ± 6.3 ^a^	39.9 ± 6.6 ^a,b^	43.3 ± 6.4 ^b^
	T1	39.0 ± 6.8	40.0 ± 5.4	41.2 ± 4.3	42.1 ± 8.2 ^a^	46.5 ± 7.1 ^a,b^	49.5 ± 6.8 ^b^
	*p*-value	0.001	0.001	0.139	0.001	0.001	0.001
	Change	2.8 ± 2.7 ^a^	2.7 ± 3.3 ^a^	1.1 ± 2.9 ^b^	5.9 ± 3.2	6.6 ± 3.8	6.1 ± 3.0
	%	8.0	7.6	2.7	16.3	17.3	14.5
FFMI (kg/m^2^)	T0	15.5 ± 2.1	15.2 ± 1.3	15.6 ± 1.1	16.0 ± 1.4	16.6 ± 1.7	16.6 ± 1.5
	T1	15.7 ± 2.2	15.6 ± 1.4	15.7 ± 1.7	16.7 ± 1.7	17.4 ± 1.8	17.4 ± 1.7
	*p*-value	0.413	0.058	0.804	0.001	0.001	0.009
	Change	0.2 ± 0.9	0.4 ± 1.0	0.1 ± 1.1	0.7 ± 0.9	0.8 ± 1.2	0.8 ± 1.0
	%	1.2	3.0	0.3	4.5	5.1	4.9
Maturity (%) *	T0	56.3	91.3	100	0	0	100
	T1	100	100	100	0	100	100

Values are expressed as means ± SDs. BMI = body mass index; FFM = fat-free mass; H = height; PhA = phase angle; R = resistance; T0 = baseline; T1 = after one year; Xc = reactance; Z = impedance vector length. Change *=* absolute difference, T1 − T0. The dependent *t*-test was used to compare one year after to baseline (intragroup), with significant *p*-values marked. * Female adolescents’ sexual maturity according to menarche occurrence and males supposed with chronological age > 13 years. Different letters in the same row and sex indicate significant differences, *p* < 0.05 (one-way ANOVA followed by Bonferroni post hoc test).

**Table 3:** Effects of initial age and time interval on raw BI data in female and male adolescents.

Female	Initial Age (T0)		Time Interval (After One Year)		Initial Age*Time Interval	
	F	*p*	F	*p*	F	*p*
PhA (°)	1.67	0.192	10.40	0.002	0.535	0.716
R/H (Ω/m)	1.42	0.244	0.37	0.544	0.370	0.689
Xc/H (Ω/m)	0.198	0.821	6.49	0.012	0.464	0.629
Z/H (Ω/m)	1.39	0.250	0.32	0.570	0.376	0.687
**Male**	**Initial Age** **(T0)**		**Time Interval** **(After One Year)**		Initial Age*Time Interval	
	**F**	***p***	**F**	***p***	**F**	***p***
PhA (°)	0.70	0.498	3.59	0.060	3.440	0.035
R/H (Ω/m)	6.63	0.002	12.96	<0.001	0.164	0.853
Xc/H (Ω/m)	4.64	0.011	5.10	0.025	2.840	0.063
Z/H (Ω/m)	6.64	0.002	12.93	<0.001	0.174	0.841

H = height; PhA = phase angle; R = resistance; Xc = reactance; Z = impedance vector length. Age (T0) represents the effect of initial age and time represents the effect after one year.

## Data Availability

The data supporting this study’s findings are available on request from the corresponding author, [JCK]. The data are not publicly available due to their containing information that could compromise the privacy of research participants.
